# Auditory and Vestibular Assessment of Patients with Type Two Diabetes Mellitus: A Case-Control Study

**DOI:** 10.22038/ijorl.2021.55334.2899

**Published:** 2021-09

**Authors:** Navid Nourizadeh, Mina Jahani, Sadegh Jafarzadeh

**Affiliations:** 1 *Sinus and Surgical Endoscopic Research Center, Faculty of Medicine, Mashhad University of Medical Sciences, Mashhad, Iran.*; 2 *Department of Otolaryngology, Faculty of Medicine, Mashhad University of Medical Sciences, Mashhad, Iran.*; 3 *Department of Audiology, School of Paramedical Sciences, Mashhad University of Medical Sciences, Mashhad, Iran. *

**Keywords:** Auditory, Diabetes, Semicircular canals, Vestibular

## Abstract

**Introduction:**

Type two diabetes mellitus may relate to auditory and vestibular dysfunction. This relationship was frequently observed in elders. The present study aimed to evaluate the auditory and vestibular function of diabetic patients and compare the results with those of a healthy adult control group.

**Materials and Methods:**

Patients were asked to complete demographic characteristics form. Moreover, fasting blood sugar, as well as hemoglobin A1C tests, were carried out on them. Both the patients and control group were evaluated using several auditory and vestibular tests including Pure Tone Audiometry (PTA), video Head Impulse Test (v-HIT), ocular Vestibular Evoked Myogenic Potential (o-VEMP), and cervical Vestibular Evoked Myogenic Potential (c-VEMP).

**Results:**

The PTA showed a significant difference in some frequencies between the two groups. These differences were minimal in lower frequencies and become greater at 8000Hz. The v-HIT was abnormal for some patients and also showed a significant difference between the two groups. The o-VEMP and c-VEMP results were normal in most patients.

**Conclusion:**

Based on the obtained results, auditory and vestibular dysfunctions are related to Diabetes. Patients with type two diabetes mellitus showed mild auditory and vestibular dysfunctions compared to the healthy control group.

## Introduction

Diabetes is one of the most prevalent metabolic disorders. Over 220 million persons have diabetes worldwide (1). Based on the World Health Organization report, this number would rise to 366 million by 2030 (1-2). The prevalence of diabetes in Iran is 7 to 15 percent of the whole population. It was significantly increased between the years 2005 and 2011 (3). Diabetes is more prevalent among women over the age of 50, retirees, urban dwellers, and people with low incomes (4-5).

Diabetes affects patients in many ways. It can cause other disorders and is related to a high mortality rate (6). It decreases the quality of life and is a high financial burden (7-9). The treatment expenses of diabetic patients are threefold that of the normal population (10).

Diabetes may affect auditory and vestibular systems as well. Many diabetic patients have vertigo, imbalance, oscillopsia, and gait abnormalities due to abnormal vestibular function (11-13). In some diabetic patients, auditory and vestibular tests show abnormal results (14,15). The relationship between diabetes and vertigo or imbalance has been evaluated in some studies (11,16-17). Many diabetic patients are elders and the aging process can greatly affect this relationship. Many older patients have auditory and vestibular dysfunction and many cases of bilateral vestibular dysfunction are elders (18). Moreover, imbalance and falling are more prevalent in elders as well as elders with diabetes (19,16,20,21).

Different tests, such as Pure Tone Audiometry (PTA), video Head Impulse Test (v-HIT), ocular Vestibular Evoked Myogenic Potential (o-VEMP), and cervical Vestibular Evoked Myogenic Potential (c-VEMP) are useful for the assessment of auditory and vestibular function. The present study aimed to assess the auditory and vestibular function of diabetic patients and compare the results with those of a healthy control group.

## Materials and Methods

**Participants**

This case-control study was performed at a general hospital in Mashhad from 2017 to 2018. The patients had type two diabetes mellitus that was diagnosed by an endocrinologist based on the American Diabetes Association standard criteria. The inclusion criteria were the presence of at least one of the following results: 1) fasting plasma glucose level ≥126 mg/dL (7.0 mmol/L), 2) 2-hour plasma glucose level ≥ 200 mg/dL (11.1 mmol/L) during a 75g oral glucose tolerance test, 3) random plasma glucose ≥ 200 mg/dL (11.1 mmol/L) accompanying classic symptoms of hyperglycemia or hyperglycemic crisis, and 4) hemoglobin A1C (HbA1c) level ≥ 6.5% (48 mmol/L).

The patients also had a negative history of any other disorders that could affect hearing or balance including presbycusis, noise-induced hearing loss or excessive noise exposure, history or family history of balance disorder. The control group was recruited from the hospital staff and some relatives of patients. They had the same gender and age as the patients. 

They didn’t have auditory, vestibular, neurologic, and metabolic disorders or any history of hearing loss, vertigo, or imbalance. All the participants were recruited using the simple random selection method and their written informed consent was obtained initially. This study was approved by the Ethics Committee of Mashhad University of Medical Sciences (IR.MUMS.fm.REC.1395.540).

**Procedure**

The demographic information of all participants including their age and gender were gathered. The medical records of the case group were checked for some extra information including duration of diabetes disorder, type of medications, last fasting blood sugar (FBS), and HbA1c test. The routine case histories for vertigo, imbalance, falling, and oscillopsia also were completed.

All participants underwent auditory and vestibular tests which were performed by an experienced audiologist under standard conditions. 

The auditory tests included PTA (MAICO, Germany), and Tympanometry (MAICO, Germany). Moreover, v-HIT (ICS impulse, Otometrics, Denmark), o-VEMP, and c-VEMP (EP25, Intracoustic, Denmark) tests were conducted on participants to evaluate five sensory organs of the vestibular functions.

In the v-HIT test, the gain was calculated for each canal. The gain was considered abnormal if it was under 0.8 for horizontal or 0.7 for vertical semicircular canals. The o-VEMP and c-VEMP responses were compared with the normal population and were considered abnormal if the waveforms were absent, late, or have more than 35% amplitude asymmetry.

**Data analysis**

The data were analyzed using SPSS software (Version 23). The Kolmogorov-Smirnov test was used to evaluate the normal distribution of data. The quantitative results were presented with the mean and standard deviation. A t-test was used to compare the results of the PTA and v-HIT tests in two groups. The results of o-VEMP and c-VEMP testing were compared in two groups by Fisher’s exact test. The Pearson correlation coefficient was used to evaluate the possible relationships between quantitative data. The relationships between other data were tested using chi-square and Fisher’s exact test. A p-value less than 0.05 (P˂0.05) was considered statistically signiﬁcant.

## Results

The data had a normal distribution. In total, 25 patients and 25 normal individuals were recruited for this study. The number of male cases was 6 and 7 in the case and control groups. The participants’ age was not significantly different in the two groups (51.32±5.01 versus 51.12±6.90; P=0.907). The mean±SD duration of diabetes disorder was 8.9±7.72 years. Injectable, oral, and both types of medications were used by 5(20%), 16(64%), and 4(16%) patients, respectively. 

The mean FBS±SD level was 71.69±171.32, and 17(68%) patients had abnormal FBS levels. HbA1c (glycohemoglobin) test was 7.87±2.14% and was abnormal in 13(52%) patients. 

Based on the clinical history of the participants, the history of vertigo, imbalance, falling, and oscillopsia was observed in 11(44%), 7(28%), 5(20%), and 2(8%) patients, respectively. 

*Auditory evaluation*

The PTA results are presented in Table 1.

**Table 1:** PTA results in case and control groups

	**Frequency**	**Case group**	**Control group**	**P-value**
Combined results of right and left ear	250 Hz	14.10±7.53	11.50±4.31	0.037
	500 Hz	12.50±6.87	10.20±3.90	0.042
	1000 Hz	10.60±8.05	8.20±3.15	0.053
	2000 Hz	14.40±8.72	12.70±7.22	0.291
	4000 Hz	24.40±16.55	20.50±16.42	0.240
	8000 Hz	36.80±21.32	24.60±22.67	0.007
Right ear	250 Hz	14.40±7.54	13.20±3.78	0.481
	500 Hz	12.00±6.45	10.60±3.62	0.349
	1000 Hz	10.40±8.28	9.00±3.53	0.443
	2000 Hz	14.00±8.78	11.40±7.00	0.253
	4000 Hz	24.00±15.34	18.60±17.17	0.247
	8000 Hz	37.60±21.36	25.40±24.66	0.068
Left ear	250 Hz	13.80±7.67	9.80±4.20	0.028
	500 Hz	13.00±7.36	9.80±4.20	0.067
	1000 Hz	10.80±7.99	7.40±2.55	0.052
	2000 Hz	14.80±8.83	14.00±7.36	0.730
	4000 Hz	24.80±17.99	22.40±15.75	0.618
	8000 Hz	36.00±21.69	23.80±20.98	0.049

The difference between the two groups was significant in 250, 500, and 8000 Hz in Combine results and 250 and 8000 Hz for the left ear. The mean difference between the two groups was small in lower frequencies (about 2-3 dB); however, in 8000 Hz it reached 12 dB. In case the patients’ hearing loss is defined for values higher than 25dB HL, a significant difference can be observed only in 8000 Hz in both right and left ears based on the results of Fisher’s exact test. Tympanometry was normal (type A) in all participants. 

**Vestibular evaluation**

The v-HIT results in the two groups are presented in Table 2.

**Table 2:** Comparison of the v-HIT results in case and control groups

**Semicircular canals**	**Ear**	**Case group (n=25)**	**Control group (n=25)**	**P-value**
Horizontal	Right	1.25±0.27	1.02±0.15	0.001
	Left	1.10±0.23	0.94±0.15	0.007
Anterior	Right	0.75±0.17	0.79±0.18	0.376
	Left	0.69±0.15	0.83±0.10	0.001
Posterior	Right	0.62±0.12	0.71±0.08	0.005
	Left	0.87±0.15	0.75±0.12	0.006

These results indicate significant differences between the two groups. However, the gain remained within normal limits in many patients.

The o-VEMP and c-VEMP responses were present in all patients. Based on the results of o-VEMP, one case had late responses in both ears and two cases were diagnosed with amplitude asymmetry. The results of the c-VEMP test indicated that two cases had late responses in both ears and one case had amplitude asymmetry. The difference between the case and control groups was not significant (P˃0.05).

There were no significant relationships among duration of diabetes disorder, FBS, or HbA1c results with PTA or v-HIT results (P˃0.05). In addition, there were no significant relationships among HbA1c (˃7%), FBS (˃130), duration of diabetes disorder (˃10 years), and the type of used medications with the history of vertigo, imbalance, falling, or oscillopsia (P˃0.05). The only significant relationship was observed between the results of HbA1c (˃7%) and the history of vertigo (P=0.028).

## Discussion

In this study, the auditory and vestibular function was evaluated in patients with type two diabetes mellitus. The results suggested mild auditory and vestibular dysfunction in some patients, compared to the healthy control group. The hearing loss was mostly observed in higher frequencies. In general, some aspects of the relationship between diabetes and hearing loss are still controversial. Some studies like the present one showed cases of hearing loss in diabetic patients (22) especially in higher frequencies (23-24), and even in patients younger than 50 years old (25). On the other hand, no relationship was observed between diabetes and the risk of hearing loss in some studies (26). The difference among various studies may be related to many different factors. Some researchers found a relationship between the duration of diabetes and the degree of hearing loss (27). This may relate to the difference and controversy between studies. Moreover, the severity of diabetes and the ways to control it could affect this relationship. The number of patients with hearing loss was higher in patients with poorly controlled diabetes than those in well-controlled groups (28). The quality of diabetes control was also unknown in different studies and could affect the study results. The degree and frequencies of hearing loss were also different in various studies; however, some studies showed similar results to those of the present study (23).

Different mechanisms were suggested for the association between diabetes and hearing loss which include oxidative stress (27), microvascular abnormalities (27-28), atrophy or thickening of the stria vascularis (25), auditory neuropathy (27,28), hearing cell dysfunctions due to electrolyte homeostasis change in endolymph (22), hearing cells damage (28), and outer hair cells damage (25). Histopathologic findings in diabetic patients also involve sclerosis of the internal auditory artery and thickening of the basilar membrane and stria vascularis (23). The changes in the auditory brainstem responses were also observed in insulin-dependent diabetic patients (29). These results were also indicative of the relationship between diabetes and vestibular dysfunction. It has been reported that diabetes could increase the risk of vestibular dysfunction (30-31). Even patients with bilateral vestibular hypofunction have a higher chance to develop diabetes (13).

In the current study, the results of the v-HIT test were abnormal in some patients and there was a significant difference between the two groups. However, the results of c-VEMP and o-VEMP were normal in the majority of cases. The increased latencies or abnormal amplitude asymmetry were observed only in few cases. Several studies evaluated the vestibular function using different tests. Vestibular hyperreflexia was the main finding in a study with the caloric test (32). These results are similar to v-HIT findings obtained in the present study. Although both of these tests evaluate the semicircular canals, the VEMP results are somehow different. In another study, more abnormal results were observed in the c-VEMP test of diabetic patients compared to their o-VEMP test (33). Abnormal c-VEMP results were also found in patients with type one diabetes with or without polyneuropathy (15). In general, abnormal and conflicting c-VEMP results were observed in some studies (30); therefore, more studies in this area are needed to explicate these discrepancies. The c-VEMP and o-VEMP tests evaluate the otolith system. Otolith dysfunction can cause vertigo, imbalance, and may lead to Benign Paroxysmal Positional Vertigo (BBPV). The prevalence of BPPV is higher in patients with diabetes (33). Otolith dysfunction also increases the recurrence risk of BPPV (34). 

The different mechanisms contributing to vestibular dysfunction include myelin degeneration of the vestibular nerve (30), saccule hair cell damage (30), metabolism changes in the inner ear (35), microvascular, and connective tissue changes (35). 

The advantage of the present study is the inclusion of adult participants to rule out aging effects. The effects of diabetes and the aging process on the auditory system can be similar (22). Moreover, the combination of diabetes and the aging process may have greater effects on the auditory and vestibular functions. The vestibular dysfunction is also more frequent in older age and diabetic patients (36).

The limitation of this study concerns the effects of diabetes on the person’s balance. In general, the risk of imbalance and falling is increased in patients with diabetes (11,30,33). However, imbalance in these patients may relate to dysfunctions of different systems and is not exclusive to vestibular dysfunction. Imbalance in patients with diabetes may relate to retinopathy (37), vestibular dysfunction (35,37), as well as reduced somatosensation and tactile sensation (38).

## Conclusion

The results of the present study suggest that there is an association between diabetes and auditory and vestibular dysfunctions. Patients with type two diabetes mellitus were suffering from mild auditory and vestibular dysfunctions compared to those in the healthy control group.
